# Notes on the Narcotics

**Published:** 1861

**Authors:** Edward Parrish

**Affiliations:** Lecturer on Pharmacy, Philadelphia


### NOTES ON THE NARCOTICS.

By EDWARD PARRISH, Lecturer on Pharmacy,

*Philadelphia.*

An experienced medical practitioner, in a recent letter to his office student, in attendance on medical lectures, advises him to acquaint himself thoroughly, during his term of study, with affections of the nervous system, as upon a thorough knowledge of these, and their treatment, his success in practice would mainly depend.

That this advice accords with the prevailing ideas of physicians, is especially obvious to the pharmaceutist, who, in comparing the recent prescriptions, on his files, with those of twenty years ago, cannot fail to notice the increased use of medicines primarily affecting the nervous system, instead of mercurials, drastic cathartics, blisters, and the like, formerly so much relied on.

From an analytical table of prescriptions which I prepared some five years ago, from my own files and those of several pharmaceutical friends, it appeared, that on an average 24 per cent. of the prescriptions examined, contained either opium, morphia, or hyosciamus, in one or other of their preparations, while the mercurials of all kinds, of which blue pill is now by far the most popular, were directed in 23 per cent. of the prescriptions; iodine and iodide of potassium in only 6 per cent., and though the files were examined with reference to the different seasons of the year, cinchona, and its alkaloids, only appeared in 9 per cent. of those written in Philadelphia. The preponderence, here shown, would probably be greatly increased in case of the preparations of opium, if the medicines dispensed without prescriptions were taken into account. Laudanum and paregoric are found in almost every dwelling, at all provided with medicines, while the great variety of carminatives and infant cordials, given so indiscriminately and so injudiciously, nearly all contain opium as their most active ingredient.

In estimating the reliance placed by physicians upon narotics, by the data above given, allowance must be made for their extensive employment as correctives of the undue influence of agents primarily affecting the secretions. This is, indeed, one of their chief uses in prescribing, and in the case of opium, especially is, perhaps, often resorted to from habit, rather than from any indication in the symptoms.

Next to opium hyosiamus is the most popular of the class of cerebral stimulants; its relaxing effect upon the bowels, secures its substitution for opium in very many instances; as an alterative, also, it shares with conium, belladonna, and stramontum, considerable reputation in appropriate combinations. The prophylactic effects of belladona, its asserted specific influence on certain eruptive diseases, and its well known usefulness is opthalmic surgery, give its extract and alkaloid, atropia, prominent positions in the class. Conium is not unfrequently prescribed with sarsaparilla and the mercurials, while stramonium is less in repute for internal use than in ointments applied to hæmorrhoids. Stramonium leaves are much employed for fumigation in asthmatic affections, a practice frequently productive of speedy relief, notwithstanding its alkaloid, daturia, is not volatile; that of conium being freely so its leaf might be supposed to furnish a much better material for the fabrication of asthmatic paper and segars. Another eligible mode of administering this class of remedies, especially those containing volatile principles, is by inhalation in connection with the vapor of water; the inconvenience of the method and the want of suitable apparatus have sometimes operated against a trial of its merits; there is no real difficulty, however, in adjusting a good inhaler, when not readily accessible, by the use of a wide-mouth bottle and glass tube.

Of all the narcotics, none has received so great an impulse, in our time, as Indian hemp. The incredible stories recorded by travelers in relation to the effects of *hashish* on the impressible natives of the East Indies, have given it a world-wide notoriety as an intoxicant, while the efforts of certain empirics, among us, who have advertised it extensively, under the plea of disinterested humanity, have drawn the attention of thousands, both in and out of the profession, to its availability in the treatment of phthisis. The extract of cannabis has, certainly, become one of the most popular, as it is one of the best of its class; its chief advantages are the exhilaration which accompanies, or rather precedes its complete narcotic effect, the remarkable control it exerts over nervous inquietude and the absence of any noxious effect, either in diminishing the appetite, checking the secretions or constipating the bowels. The quality of the preparation is so varied, that physicians are often quite uncertain what dose to prescribe; some specimens seem nearly inert, while of others half a grain is a powerful dose, and a grain has produced alarming symptoms. It is always best for the physician to assure himself beforehand on this head. It should be of a bright green color, heavy narcotic odor, and entirely soluble in alcohol. Squire's London manufacture is esteemed the best. In prescribing extract of cannabis, in liquid form, the most approved combination is with carbonate of potassa, which renders the extract soluble in water, otherwise, dissolved in alcohol, it readily separates on being added to aqueous and even mucilaginous preparations. I have met with one case only in which extract of cannabis produced those extraordinary hallucinations to which I have referred as being ascribed to *hashish* in the East. This patient, a consumptive, was often possessed with an idea of his own duality, seeing horrid visions of detached portions of himself. He seemed to be continually haunted by the presence of an imperious director, on whose behalf his most trifling acts were performed. At times, even in the act of expectoration, he believed himself the agent of another for whose sufferings he felt the keenest sympathy.

The very extensive use made of lobelia and sanguinaria in this country, would, I think, surprise our transatlantic calaborers. The combination, in these remedies, of narcotic with emetic and diaphoretic properties, fits them for a great variety of indications, though they seem to me by no means so safe and harmless as some rather adventurous prescribers seem to suppose. A plant of the famous solanacæ family, to which belladonna, stramonium, and hyosciamus belong, yields us the alternative narcotic dulcamara, of which we have only two preparations in the Pharmacopœia—the decoction and aqueous extract, neither of them very eligible or apparently satisfactory to the profession.

One very prominent element in the popularity of a remedy is its pharmaceutical eligibility, and there is little doubt that the neglect into which many valuable drugs have fallen is due chiefly to their not being presented to the practitioner in an eligible shape. Since the general abandonment of the crude and inelegant class of infusions and decoctions, there has been a manifest tendency to employ more concentrated and reliable preparations; but the Pharmacopœia, never in advance of the demands of the profession, has given no formulas in the cases of many of the most valuable. That this may be partially remedied in the edition now under revision by the eminently judicious and conservative committee appointed in Washington some eighteen months since, is greatly to be desired.

Fluid extracts are now loudly called for on all hands. That many of this class of liquid preparations originated, and have come into use through the commercial enterprise and activity of certain manufacturing pharmaceutists, not amenable to the careful scrutiny of the profession and the public, and whose processes are too uniform to be properly applicable to every drug, is no argument against the excellent scheme of concentrating liquid preparations to such point as that their dose shall have the same relation in each case to that of the drug from which derived. Modern pharmacy, with a full appreciation of the composition of almost every drug known to the profession, is now prepared to adapt solvents to each, which will fully extract its medicinal principles, discarding those which are inert, and, by processes of evaporation within the reach of every apothecary, to reduce these to concentrated and permanent forms. The alcohol is sometimes an objection in tinctures, from therapeutic incompatibility, while in syrups, which are always given in comparatively large doses, the sugar is equally objectionable. Fluid extracts, besides the advantage of correspondence of dose with that of the drug, contain a minimum of either of these antiseptic ingredients. They are, however, best adapted to drugs the doses of which are not very small, and I can see no advantage, for internal use, in more concentrated liquid preparations of the powerful narcotics than their tinctures already furnish, especially as the solid extracts are so easily incorporated with extemporaneous mixtures. The imported preserved juices are not recommended so much for greater concentration as for their careful preparation directly from the fresh expressed juice of the plant before it has been altered by any processes of drying and subsequent treatment with menstrua.

In this connection, it may not be out of place to notice some facts in regard to the preparations of opium, of especial advantage in those cases in which Laudanum is contraindicated by its nauseating effects and the disagreeable symptoms as its effects are passing off. McMunn's elixir has attained a place among the popular preparations of opium from the absence of these objectionable qualities, and the only obstacle to its use by physicians lies in its being a nostrum, the mode of preparation of which is carefully concealed. The prevailing opinion among pharmaceutists appears to be that this is an aqueous preparation, to which, after its completion, a very little alcohol is added to prevent decomposition, and, on this principle, numerous elixirs of opium are offered, though, as might be expected, McMunn's still takes precedence. The aqueous extract is a preparation well worthy of a fair trial in competition with the drug itself, or its tinctures; on theoretical grounds, it gives promise of good results. I have met with practitioners who unhesitatingly give the preference to black drop and acetated tincture of opium, which they recommend as greatly preferable to the ordinary liquid preparation, while certain confirmed opium-drunkards give their testimony in the same direction, asserting that these preparations, containing morphia as acetate, produce less emaciation from their habitual use than those containing the natural salt, meconate of morphia. On the other hand, I have lately imported from London an elegant preparation called solution of bi-meconate of morphia, resembling McMunn's elixir, though still more completely deprived of odor, and apparently quite free from the usual objections to laudanum.

What is the dose of the morphia salts? One-eighth of a grain, say the older books and prescriptions; one-third of a grain, say recent authorities; one grain, say now a great many physicians, not only here but elsewhere. Formerly I hesitated to compound a prescription calling for this dose, now it is so frequently prescribed and administered, that we begin to regard it as safe, and cannot help setting down the fact as an offset to the asserted lessening of doses to meet the demand of the times.—*Med. Reporter.*

